# Leukocyte telomere length in relation to the risk of Barrett's esophagus and esophageal adenocarcinoma

**DOI:** 10.1002/cam4.810

**Published:** 2016-07-06

**Authors:** E. Christina M. Wennerström, Rosa A. Risques, Donna Prunkard, Carol Giffen, Douglas A. Corley, Liam J. Murray, David C. Whiteman, Anna H. Wu, Leslie Bernstein, Weimin Ye, Wong‐Ho Chow, Thomas L. Vaughan, Linda M. Liao

**Affiliations:** ^1^ Division of Cancer Epidemiology and GeneticsNational Cancer InstituteBethesdaMaryland; ^2^ Department of Epidemiology ResearchStatens Serum InstitutCopenhagenDenmark; ^3^ Department of PathologyUniversity of WashingtonSeattleWashington; ^4^ Information Management ServicesBethesdaMaryland; ^5^ Division of Research and Oakland Medical CenterKaiser PermanenteNorthern California, OaklandCalifornia; ^6^ Centre for Public HealthQueen's UniversityBelfastUnited Kingdom; ^7^ QIMR Berghofer Medical Research InstituteBrisbaneAustralia; ^8^ School of Population HealthUniversity of QueenslandBrisbaneAustralia; ^9^ Department of Preventive MedicineKeck School of Medicine, University of Southern California/Norris Comprehensive Cancer CenterLos AngelesCalifornia; ^10^ Division of Cancer EtiologyDepartment of Population ScienceBeckman Research Institute, City of HopeDuarteCalifornia; ^11^ Department of Medical Epidemiology and BiostatisticsKarolinska InstitutetStockholmSweden; ^12^ UT MD Anderson Cancer CenterHoustonTexas; ^13^ Division of Public Health SciencesFred Hutchinson Cancer Research CenterSeattleWashington

**Keywords:** Barrett's esophagus, esophageal Adenocarcinoma, epidemiology, telomere

## Abstract

Chronic inflammation and oxidative damage caused by obesity, cigarette smoking, and chronic gastroesophageal reflux disease (GERD) are major risk factors associated with Barrett's esophagus (BE) and esophageal adenocarcinoma (EAC). EAC has been increasing the past few decades, and early discovery and treatment are crucial for survival. Telomere shortening due to cell division and oxidative damage may reflect the impact of chronic inflammation and could possibly be used as predictor for disease development. We examined the prevalence of shorter leukocyte telomere length (LTL) among individuals with GERD, BE, or EAC using a pooled analysis of studies from the Barrett's and Esophageal Adenocarcinoma Consortium (BEACON). Telomere length was measured in leukocyte DNA samples by Q‐PCR. Participants included 1173 patients (386 with GERD, 384 with EAC, 403 with BE) and 736 population‐based controls. The association of LTL (in tertiles) along the continuum of disease progression from GERD to BE to EAC was calculated using study‐specific odds ratios (ORs) and 95% confidence intervals (CIs) from logistic regression models adjusted for potential confounders. Shorter LTL were less prevalent among GERD patients (OR 0.57; 95% CI: 0.35–0.93), compared to population‐based controls. No statistically significant increased prevalence of short/long LTL among individuals with BE or EAC was observed. In contrast to some earlier reports, our findings add to the evidence that leukocyte telomere length is not a biomarker of risk related to the etiology of EAC. The findings do not suggest a relationship between LTL and BE or EAC.

## Introduction

Oxidative stress can cause large intragenic deletions, as well as chromosomal loss and chromosomal instability 1. Most areas of the chromosome have built in repair systems to manage oxidative damage. However, telomeres, the repetitive DNA that cap the end of chromosomes, are especially susceptible to damage due to relative inefficient repair of single‐strand breaks. This leads to accelerated telomere shortening, which adds to the constant rate of telomere shortening by cell division 1, 2. Factors associated with oxidative stress and chronic inflammation, such as obesity and smoking, have been associated with increased telomere shortening 3.

Gastroesophageal reflux disease (GERD) and Barrett's esophagus (BE) are both major risk factors in the development of esophageal adenocarcinoma (EAC), which has been rapidly increasing in incidence in the Western world 4. GERD is caused by abnormal relaxation of the lower esophageal sphincter that normally holds the stomach closed and prevents gastric acid from entering the esophagus. In GERD, exposure of the esophageal epithelium to bile and gastric acid leads to inflammation and chronic mucosal damage; a minority of individuals, estimated at 8–15%, develop BE 5, 6. In BE, the squamous epithelium of the esophagus is replaced with metaplastic columnar epithelium 7. A small proportion of individuals with BE progress to EAC, with an estimated incidence of EAC among persons with nondysplastic BE of 0.3% per year 4. Furthermore, the major risk factors associated with BE and EAC, such as obesity, GERD, and cigarette smoking, contribute to chronic inflammation and are likely to contribute to telomere length shortening 3, 8.

Several studies have, however, reported associations between telomere length and esophageal cancer, with somewhat conflicting results 9, 10, 11.

In this analysis, we evaluated leukocyte telomere length (LTL) in three sets of cases: GERD, BE, and EAC, using seven studies from the Barrett's and EAC Consortium (BEACON, http://beacon.tlvnet.net/). We hypothesized that the prevalence of LTL shortening would be higher among those with more severe disease compared to population‐based controls.

## Material and Methods

### Study population

Data and samples from seven studies in the international BEACON consortium were included in this analysis. Individual information on age, gender, race, body mass index (BMI), smoking, alcohol, DNA concentration, and extraction method was collected from each study. Two studies contributed samples from individuals with GERD: the Study of Reflux Disease (SRD) based in western Washington, USA 12 and the Factors Influencing the Barrett's/Adenocarcinoma Relationship (FINBAR) study, based in Ireland 13. SRD, FINBAR, the Study of Digestive Health (SDH), based in Brisbane, Australia 14; and the Epidemiology and Incidence of Barrett's Oesophagus (EIBO) study, based in the Kaiser Permanente Northern California population, USA 15 provided samples from individuals diagnosed with BE. Four studies contributed samples from individuals diagnosed with EAC: the FINBAR study; the Los Angeles Multi‐ethnic (LAM) Study 16; Australian Cancer Study (ACS) 17; and the Swedish Esophageal and Cardia Cancer (SECC) study 18.

Restricted to Whites only, we selected 400 individuals with GERD, 404 individuals with BE, 384 individuals with EAC, and 749 population‐based controls.

Controls were selected from the same source population from which the cases arose and were frequency matched to cases by gender and age in each study. In total, 386 GERD individuals, 403 BE individuals, 384 EAC individuals, and 736 controls were included in our analysis (Table S1). In total 173 samples were excluded from our analysis. A total of 17 samples had insufficient amounts of DNA, 121 samples either had missing information on DNA extraction method or DNA was extracted with various methods within the study population. Furthermore, 35 cardia stomach cancer cases were previously misclassified as EAC cases, these cases were excluded from the analysis when this was discovered.

IRB approval was obtained for each of the included studies.

### Telomere length measurements

Four different methods were used to extract leukocyte DNA from whole blood among the selected studies: 5‐Prime (5‐Prime, Hilden, Germany) was used in the SRD study; Gentra Puregene DNA purification kit (Qiagen, Hilden, Germany) was used in the FINBAR, EIBO, and SECC studies; the salting out method 19 was used in the SDH and ACS studies; and the QIAamp DNA Blood kit (Qiagen) was used in the LAM study (Table 1).

**Table 1:** Study characteristics among populations‐based controls by study population and DNA extraction method

	*N*	Gender	Age	BMI	Smoking ever (%)	LTL median (IQR)	
		Male (%)	Mean years (SD)	Mean kg/m^2^ (SD)			
Study [reference number]							
The Study of Reflux Disease (SRD) 12	100	60 (60.0)	51.7 (12.5)	27.7 (4.7)	48 (48.0)	1.03	(0.92–1.21)
The Factors Influencing the Barrett's/Adenocarcinoma Relationship (FINBAR) Study 13	91	82 (90.1)	61.7 (13.8)	27.1 (4.0)	55 (60.8)	0.80	(0.70–0.96)
The Study of Digestive Health (SDH) 14	104	75 (72.1)	61.3 (11.0)	27.4 (5.1)	56 (53.9)	0.87	(0.77–0.97)
The Epidemiology and Incidence of Barrett's Oesophagus (EIBO) Study 15	100	71 (71.0)	64.0 (10.1)	28.7 (4.6)	59 (59.0)	0.98	(0.89–1.11)
The Los Angeles Multi‐ethnic (LAM) Study 16	53	47 (88.7)	61.0 (9.8)	25.9 (5.2)	33 (62.3)	0.71	(0.67–0.84)
The Australian Cancer Study (ACS) 17	242	225 (92.9)	63.3 (9.5)	27.7 (4.7)	148 (61.2)	0.94	(0.84–1.06)
The Swedish Esophageal and Cardia Cancer (SECC) Study 18	46	40 (87.0)	66.6 (8.2)	23.5 (1.9)	25 (54.4)	0.87	(0.76–0.99)
DNA extraction method (company/reference)							
Gentra Puregene DNA purification kit (Qiagen, Hilden, Germany)^a^	237	193 (81.4)	63.6 (11.5)	27.1 (4.4)	139 (59.1)	0.91	(0.77–1.03)
Protein Salting Out 19, b	346	300 (86.7)	62.7 (10.0)	27.6 (4.8)	204 (59.0)	0.92	(0.80–1.03)
Qiagen QIAamp DNA Blood kit (Qiagen)^c^	53	47 (88.7)	61.0 (9.8)	25.6 (5.2)	33 (62.3)	0.71	(0.67–0.84)
5‐Prime (5‐Prime, Hilden, Germany)^d^	100	60 (60.0)	51.7 (12.5)	27.5 (4.7)	48 (48.0)	1.03	(0.92–1.21)
Total	736	604 (82.1)	61.4 (11.5)	27.3 (4.7)	425 (57.8)	0.92	(0.79–1.04)

Extraction method used in ^a^FINBAR, EIBO, and SECC study; ^b^SDH and ASC study; ^c^LAM study; ^d^SRD study. LTL, leukocyte telomere length; IQR, interquartile range; SD, standard deviation; BMI, body mass index.

LTL was measured by quantitative PCR (q‐PCR) at the Cytometry and Telomere Center, Department of Pathology, University of Washington, using the method described by Cawthon 20. In brief, for each sample, two PCRs were performed: the first one to amplify the telomeric DNA and the second one to amplify a single‐copy control gene (36B4, acidic ribosomal phosphoprotein PO). This provided an internal control to normalize the starting amount of DNA. A four‐point standard curve (twofold serial dilutions from 5 to 0.625 ng of DNA) was included in all PCRs to allow the transformation of Ct (cycle threshold) into nanograms of DNA. The telomere PCR reactions are further described in Table S2. The PCR amplification raw data were exported to an in‐house software that aligned all the amplification plots to a baseline height and calculated the Ct based on a fluorescence threshold set at the beginning of the exponential phase of the plots. The baseline height and fluorescence threshold had constant values for all the PCRs in the analysis. All samples were analyzed in triplicate and the median value of Cts was used for subsequent calculations. Cts were converted into nanograms of DNA using standard curves. The amount of telomere DNA was divided by the amount of control‐gene DNA, producing a relative measurement of the LTL. Each experiment included 24 samples and 4 laboratory provided DNA controls. The laboratory DNA controls were used to normalize between experiments and to assess variability. The number of cases and controls were balanced in each experiment, to ensure that cases and controls were treated equally and thereby comparable. As specimens came from seven different study populations, reproducibility was tested intra‐ and interstudy. Reproducibility intrastudy was analyzed by repeating a subset of the samples analyzed within each study (12–50, depending on study size) and calculating the coefficient of variation (CV) between the first and the second measurement. The mean CV for each study was as follows: SRD, 7.4%; EIBO, 4.7%; FINBAR, 5.3%; ACS 5.3%; SDH, 4.5%; LAM, 4.6%; and SECC, 5.7%. Reproducibility interstudy was analyzed by repeating 3–5 samples randomly selected from each study, for a total of 24 samples. The mean CV was 6.4%, which is in agreement with the variability reported by other groups 21. Potential outlier samples with extremely long or short LTL were repeated to confirm measurements. All laboratory personnel were blinded to case/control status in all assays conducted.

### Statistical analysis

The association of LTL with GERD, BE, and EAC was calculated using a two‐step analytic approach. First, the association between LTL and each outcome was investigated separately in each of the included studies. LTL was treated both as a continuous variable and categorized as tertiles (with T1, the longest, serving as reference) based on the distribution among population‐based controls. We used logistic regression modeling to calculate study‐specific odds ratios (ORs) and 95% confidence intervals (CIs). Site‐specific ORs were pooled together using individual results from each relevant BEACON study. The model was minimally adjusted for gender, age (continuous), and method of DNA extraction. Further in the fully adjusted model for smoking status (ever or never smoking), BMI (categorized into; <18.5, 18.5–24.9, 25–29, 30–34.9, 35–39.9, and >40 kg/m^2^) and alcohol consumption (ever or never drinking), and a subsequent analysis also including years of smoking (categorized into; never smoking, 0.5–10, 10–20, 20–30, 30–40, and >40 years of smoking). To evaluate possible effect modification, we conducted sensitivity analyses by mean age at recruitment (<60 years or >60 years), gender, BMI (<30 kg/m^2^ or >30 kg/m^2^), smoking status (ever or never smoking), and alcohol intake (ever or never drinking).

## Results

We confirmed earlier findings that DNA extraction method had an effect on LTL 22. Specifically, QIAamp‐extracted DNA had generally shorter LTL compared to DNA extracted with salting out methods. This difference was observed among controls in our study as well; for this reason, DNA extraction method was included as a covariate in both the minimally and fully adjusted models (Table 1).

### Characteristics of study populations

Seven study populations were included from the BEACON consortium (Table 1). Characteristics by study population and DNA extraction method are given in Table 1. In brief, more than 80% of the study participants were men. The mean age of all participants was 61 years. More than half of the participants were smokers and most were overweight (mean BMI of 27.3 kg/m^2^). LTL was negatively correlated with male gender (*r* = −0.12, *P* = 0.001), increased age (*r* = −0.31, *P* < 0.001), and positively correlated with alcohol use (*r* = 0.10, *P* = 0.006). LTL was negatively correlated with BMI (*r* = −0.02, *P* = 0.51), although not statistically significant. We found no correlation between LTL and ever/never smoking (*r* = −0.0396, *P* = 0.28). However, years of smoking was correlated with LTL (*r* = 0.10, *P* = 0.005). Subsequently, including years of smoking in our model did not change the results notably in the main analysis (data not shown). The longest median LTL was observed among those with GERD (median 0.96, interquartile range [IQR]: 0.83–1.16), followed by BE (median 0.94, IQR: 0.82–1.08) and EAC (median 0.88, IQR: 0.76–1.02) (Table S3).

### LTL and GERD

Compared to population controls, a decreased risk of GERD was observed among those with short LTL (T3 vs. T1), OR 0.57 (95% CI: 0.35–0.93) (Table 2). Very little difference was observed between the minimally adjusted and the fully adjusted models. In analyses stratified by gender and age, the associations among men and patients less than 60 years of age were similar to the overall results, respectively, OR 0.38 (95% CI: 0.21–0.69) and OR 0.41 (95% CI: 0.20–0.83) (Table 3).

**Table 2:** Leukocyte telomere length (LTL) and prevalence of GERD, BE, and EAC minimally and fully adjusted

				Pooled analysis	
	LTL	Case	Control	OR (95% CI)^a^	OR (95% CI)^b^
GERD versus population‐based controls	1st (Long)	177	77	1 [Reference]	1 [Reference]
Studies included; SRD, FINBAR	2nd	112	49	0.92 (0.59–1.45)	0.93 (0.58–1.47)
	3rd (Short)	97	65	0.57 (0.35–0.93)	0.57 (0.34–0.93)
	Continuous			0.38 (0.16–0.91)	0.34 (0.14–0.86)
	*P*_trend_			0.03	0.02
BE versus population‐based controls	1st (Long)	153	142	1 [Reference]	1 [Reference]
Studies included; SRD, FINBAR, SDH, EIBO	2nd	139	126	0.98 (0.69–1.38)	0.95 (0.67–1.36)
	3rd (Short)	111	127	0.75 (0.51–1.08)	0.74 (0.51–1.09)
	Continuous			0.65 (0.32–1.32)	0.58 (0.28–1.20)
	*P*_trend_			0.23	0.14
EAC versus population‐based controls	1st (Long)	106	119	1 [Reference]	1 [Reference]
Studies included; FINBAR, LAM, ACS, SECC	2nd	124	143	0.96 (0.67–1.38)	0.92 (0.63–1.34)
	3rd (Short)	154	170	1.01 (0.69–1.46)	0.96 (0.65–1.42)
	Continuous			1.37 (0.62–3.01)	1.34 (0.59–3.06)
	*P*_trend_			0.44	0.49
BE versus GERD	1st (Long)	75	177	1 [Reference]	1 [Reference]
Studies included; SRD, FINBAR	2nd	61	112	1.25 (0.81–1.95)	1.21 (0.77–1.89)
	3rd (Short)	64	97	1.48 (0.91–2.40)	1.42 (0.86–2.33)
	Continuous			1.85 (0.76–4.51)	1.83 (0.74–4.53)
	*P*_trend_			0.18	0.19

OR, odds ratio; CI, confidence interval; GERD, gastroesophageal reflux; BE, Barrett's esophagus; EAC, esophageal adenocarcinoma; FINBAR, the Factors Influencing the Barrett's/Adenocarcinoma Relationship Study; EIBO, the Epidemiology and Incidence of Barrett's Oesophagus study; SECC, the Swedish Esophageal and Cardia Cancer study; ACS, the Australian Cancer Study; SDH, the Study of Digestive Health; LAM, the Los Angeles Multi‐ethnic Study; SRD, the Study of Reflux Disease.

a Minimally adjusted for age, gender, and DNA extraction method.

b Fully adjusted for age, gender, ever smoking, BMI, alcohol, and DNA extraction method.

**Table 3:** Leukocyte telomere length (LTL) and prevalence of GERD, BE, and EAC minimally adjusted, stratified by gender and age

		Men			Women			<60 years old			≥60 years old		
	LTL	Control	Case	OR (95% CI)^a^	Control	Case	OR (95% CI)^a^	Control	Case	OR (95% CI)^b^	Control	Case	OR (95% CI)^b^
GERD versus population‐based controls	1st (Long)	48	114	1 [Reference]	29	63	1 [Reference]	55	133	1 [Reference]	22	44	1 [Reference]
Studies included; SRD, FINBAR	2nd	37	77	0.74 (0.43–1.28)	12	35	1.53 (0.66–3.53)	29	58	0.76 (0.43–1.33)	20	54	1.35 (0.64–2.85)
	3rd (Short)	54	70	0.38 (0.21–0.69)	8	27	1.82 (0.66–4.97)	24	31	0.41 (0.20–0.83)	41	66	0.81 (0.41–1.62)
	Continuous			0.18 (0.06–0.54)			2.24 (0.44–11.3)			0.21 (0.06–0.67)			0.71 (0.19–2.58)
	*P*_trend_			0.01			0.33			0.01			0.60
BE versus population‐based controls	1st (Long)	86	103	1 [Reference]	56	50	1 [Reference]	88	95	1 [Reference]	54	58	1 [Reference]
Studies included; SRD, FINBAR, SDH, EIBO.	2nd	96	106	0.91 (0.60–1.37)	30	33	1.14 (0.59–2.19)	60	58	0.85 (0.52–1.37)	66	81	1.16 (0.70–1.91)
	3rd (Short)	106	89	0.68 (0.44–1.04)	21	22	1.05 (0.47–2.32)	39	31	0.67 (0.37–1.21)	88	80	0.86 (0.53–1.39)
	Continuous			0.71 (0.32–1.58)			0.48 (0.11–2.11)			0.51 (0.18–1.39)			0.85 (0.32–2.24)
	*P*_trend_			0.40			0.33			0.19			0.74
EAC versus population‐based controls	1st (Long)	109	100	1 [Reference]	10	6	1 [Reference]	60	55	1 [Reference]	59	51	1 [Reference]
	2nd	131	110	0.91 (0.62–1.32)	12	14	2.28 (0.60–8.69)	51	40	0.87 (0.61–1.36)	92	84	1.04 (0.64–1.68)
	3rd (Short)	154	139	0.97 (0.65–1.43)	16	15	1.73 (0.45–6.59)	42	42	1.12 (0.60–2.08)	128	112	1.02 (0.64–1.63)
Studies included; FINBAR, LAM, ACS, SECC	Continuous			1.37 (0.60–3.14)			1.34 (0.10–18.8)			1.34 (0.38–4.77)			1.51 (0.56–4.10)
	*P*_trend_			0.45			0.83			0.65			0.42
BE versus GERD	1st (Long)	114	49	1 [Reference]	63	26	1 [Reference]	133	58	1 [Reference]	44	17	1 [Reference]
Studies included; SRD, FINBAR	2nd	77	47	1.65 (0.96–2.83)	35	14	0.76 (0.34–1.72)	58	32	1.29 (0.74–2.23)	54	29	1.50 (0.71–3.17)
	3rd (Short)	70	52	2.16 (1.19–3.91)	27	12	0.65 (0.26–1.67)	31	20	1.52 (0.76–3.06)	66	44	1.92 (0.92–4.00)
	Continuous			6.42 (2.01–20.5)			0.24 (0.06–1.06)			1.92 (0.63–5.83)			3.62 (0.81–16.1)
	*P*_trend_			0.01			0.06			0.25			0.09

LTL, leukocyte telomere length; OR, odds ratio; CI, confidence interval; GERD, gastroesophageal reflux; BE, Barrett's esophagus; EAC, esophageal adenocarcinoma; FINBAR, the Factors Influencing the Barrett's/Adenocarcinoma Relationship study; EIBO, the Epidemiology and Incidence of Barrett's Oesophagus study; SECC, the Swedish Esophageal and Cardia Cancer study; ACS, the Australian Cancer study; SDH, the Study of Digestive Health; LAM, the Los Angeles Multi‐ethnic Study; SRD, the Study of Reflux Disease.

a Adjusted for age and DNA extraction method.

b Adjusted for gender and DNA extraction method.

### LTL and BE

Compared to patients with GERD, those with BE were more likely to have short telomere length (T3 vs. T1) (OR 1.48 [95% CI: 0.91–2.40]); this was, however, not statistically significant (Table 2). When stratified by gender, an increased risk of BE was observed among men with short telomere length (T3 vs. T1) when compared to GERD controls (OR 2.16 [95% CI: 1.19–3.19]), but not compared with population‐based controls (Table 3). Interestingly, a twofold increased probability was also observed among ever smokers, alcohol consumers, and those with a BMI <30 kg/m^2^ (Table S4).

### LTL and EAC

Compared to populations‐based controls we observed no associations between telomere length and EAC overall or when stratified by age, gender, BMI, smoking, or alcohol drinking (Tables 3 and S4).

## Discussion

Until recently, telomere length measurement in large epidemiologic studies has been limited. Telomere shortening has been observed in precursor lesions for various cancers, and therefore suggested to be a suitable biomarker for cancer incidence and mortality 23, 24. Associations with telomere length have been reported for cancers of the lung, bladder, colorectal, breast, and the digestive system 25, 26, 27, 28. This study explored the possibility of using telomere length as an early prognostic biomarker to identify individuals at risk of EAC. This study was positioned to provide insight into a possible telomere length gradient in several related stages in the progression from reflux disease to BE and further on to EAC.

In this pooled analysis, we included DNA from seven study populations and almost 2000 participants, from the BEACON consortium.

Our findings suggest that short LTL was associated with GERD compared to population‐based controls. Although EAC is thought to progress from a state of chronic GERD or BE, we could not confirm the previously reported chromosomal instability and decrease in telomere length associated with BE or EAC 9, 29, 30. Some of the previous studies were based on telomere length measured in tissue and not peripheral blood, and therefore might not be directly comparable. Disease progression has been suggested to explain why short LTL among BE patients is associated with an increased risk of EAC. Alternatively, LTL shortening could share the same causal risk factors as BE and EAC, for example, inflammation 31, 32. Another explanation for inconsistent findings could be due to timing of telomere shortening. Shortening occurring early on in neoplastic progression could result in LTL being highly variable among BE patients 29.

Compared to individuals with GERD, short LTL was associated with BE, although this was statistically significant only among men. Gender differences associated with telomere length has previously been shown; however, the limited number of female patients in this study made it difficult to ascertain potential differences in associations between the genders 33, 34. Furthermore, generalizability to populations other than Caucasians may be limited.

A major strength of this study is its cross‐sectional design with the large number of well‐characterized samples from multiple studies within the BEACON consortium. By pooling studies within the BEACON consortium, we were able to utilize studies which contribute valuable information on major risk factors associated with BE and EAC, such as obesity, smoking status, and GERD. Although we controlled for confounding in our multivariable analysis, potential unmeasured confounders or residual confounding exists as insufficient information about other factors that may be associated with healthy aging and telomere length might be lacking. We could not confirm LTL shortening to be associated with alcohol use and BMI; however, this is a fairly complicated and complex association that has been suggested to mainly be related to age 31, 32, 33.

Previous studies have demonstrated associations between LTL and markers of inflammation 9, 23, 31, 32, 33, 35. Unfortunately, a limitation of our study is that we did not have measurements of inflammatory biomarkers in our study and instead rely on self‐reported lifestyle risk factors such as obesity, smoking, and alcohol consumption to serve as surrogates of inflammation. Chronic inflammation is a major influence in the development of disease in individuals with GERD, BE, and EA, however, a recent study investigating several inflammatory biomarkers in patients with BE reported limited correlation with LTL length 33.

An additional limitation of our study design was the inherent differences in DNA extraction and storage among participating studies. The quality of DNA from various methods of DNA extraction has been proven to impact the measurement of LTL 22, 36. To diminish this problem in this analysis, we selected samples using the same method of DNA purification within each study, and included DNA extraction method as a covariate in all models. However, residual confounding by extraction method is likely to be present and cannot be excluded. The exclusion of 173 samples from our study could have led to some potential bias, but this is not likely as the reasons samples were excluded (e.g., insufficient amounts of DNA, missing DNA extraction method) are not associated with LTL or case status.

Although we detected longer LTL among individuals with GERD, LTL was not consistently associated with BE or EA. Our findings suggest that short LTL is not associated with progression from GERD to BE to EAC. Therefore, we cannot suggest LTL to be a suitable early prognostic biomarker for EAC in prevention and diagnostics. These results underline the complexity of LTL and cancer risk. Although we evaluated results for two possible EAC precursors, GERD and BE, we do not have a lifetime history of study participants. Therefore, repeated LTL measurement in a longitudinal setting covering the entire lifespan and disease progression could provide further insight on telomere dynamics in EAC disease progression.

## Conflict of Interest

Nothing to declare. All authors critically revised the manuscript for intellectual content and approved the final draft submitted.

## Supporting information


**Table S1.** Study sample flowchart. Study abbreviation: FINBAR, the Factors Influencing the Barrett's/Adenocarcinoma Relationship study; EIBO, the Epidemiology and Incidence of Barrett's Oesophagus study; SECC, the Swedish Esophageal and Cardia Cancer study; ACS, the Australian Cancer study; SDH, the Study of Digestive Health; LAM, the Los Angeles Multi‐ethnic Study; SRD, the Study of Reflux Disease.
**Table S2.** qPCR reaction. All PCR reactions were set up with a QIAgility pipetting robot (Qiagen, Hilden, Germany) and performed in 100‐well using Rotor Gene Q (Qiagen). Each reaction included 1× Rotor Gene Sybrgreen PCR master mix (Qiagen), 2.5 ng of DNA, 500 nmol/L primer, and 1 uM control‐gene primer. In addition, 400 nmol/L of a passive HEX‐labeled oligo was included in all reactions as a passive reference dye. The Rotor Gene software (Qiagen) was used to normalize the Sybrgreen intensity to the HEX passive reference dye.
**Table S3.** Study characteristics of GERD, BE, and EAC cases by study. The following studies used Gentra Puregene DNA purification kit (Qiagen, Hilden, Germany): FINBAR, EIBO, and SECC. ACS and SDH used Protein Salting Out method. In SRD study 5‐Prime (5‐Prime, Hilden, Germany) was used and in LAM study Qiagen QIAamp DNA Blood kit (Qiagen) was used. Abbreviations: FINBAR, the Factors Influencing the Barrett's/Adenocarcinoma Relationship study; EIBO, the Epidemiology and Incidence of Barrett's Oesophagus study; SECC, the Swedish Esophageal and Cardia Cancer study; ACS, the Australian Cancer study; SDH, the Study of Digestive Health; LAM, the Los Angeles Multi‐ethnic Study; SRD, the Study of Reflux Disease.
**Table S4.** Leukocyte telomere length and prevalence of GERD, BE, and EAC stratified by smoking, obesity, and alcohol consumption. Odds ratios and 95% confidence interval for leukocyte telomere length (LTL) (1^st^, 2^nd^, and 3^rd^ tertile) with risk of gastroesophageal reflux (GERD), Barrett's esophagus (BE), and esophageal adenocarcinoma (EAC), stratified by smoking (ever smokers and nonsmokers) BMI (<30 kg/m^2^ and >30 kg/m^2^), and alcohol consumption (consumers and nonconsumers). The analysis was minimally adjusted for age, gender, and DNA extraction method.
