# Supplementary material for: Leukocyte telomere length in relation to the risk of Barrett's esophagus and esophageal adenocarcinoma
Source: Cancer Med. 2016 Jul 6;5(9):2657–65. doi: 10.1002/cam4.810 (PMC5055192; doi:10.1002/cam4.810)
Supplement: Supplementary file 1 — Table S1. Study sample flowchart. Study abbreviation: FINBAR, the Factors Influencing the Barrett's/Adenocarcinoma Relationship study; EIBO, the Epidemiology and Incidence of Barrett's Oesophagus study; SECC, the Swedish Esophageal and Cardia Cancer study; ACS, the Australian Cancer study; SDH, the Study of Digestive Health; LAM, the Los Angeles Multi‐ethnic Study; SRD, the Study of Reflux Disease. Table S2. qPCR reaction. All PCR reactions were set up with a QIAgility pipetting robot (Qiagen, Hilden, Germany) and performed in 100‐well using Rotor Gene Q (Qiagen). Each reaction included 1× Rotor Gene Sybrgreen PCR master mix (Qiagen), 2.5 ng of DNA, 500 nmol/L primer, and 1 uM control‐gene primer. In addition, 400 nmol/L of a passive HEX‐labeled oligo was included in all reactions as a passive reference dye. The Rotor Gene software (Qiagen) was used to normalize the Sybrgreen intensity to the HEX passive reference dye. Table S3. Study characteristics of GERD, BE, and EAC cases by study. The following studies used Gentra Puregene DNA purification kit (Qiagen, Hilden, Germany): FINBAR, EIBO, and SECC. ACS and SDH used Protein Salting Out method. In SRD study 5‐Prime (5‐Prime, Hilden, Germany) was used and in LAM study Qiagen QIAamp DNA Blood kit (Qiagen) was used. Abbreviations: FINBAR, the Factors Influencing the Barrett's/Adenocarcinoma Relationship study; EIBO, the Epidemiology and Incidence of Barrett's Oesophagus study; SECC, the Swedish Esophageal and Cardia Cancer study; ACS, the Australian Cancer study; SDH, the Study of Digestive Health; LAM, the Los Angeles Multi‐ethnic Study; SRD, the Study of Reflux Disease. Table S4. Leukocyte telomere length and prevalence of GERD, BE, and EAC stratified by smoking, obesity, and alcohol consumption. Odds ratios and 95% confidence interval for leukocyte telomere length (LTL) (1st, 2nd, and 3rd tertile) with risk of gastroesophageal reflux (GERD), Barrett's esophagus (BE), and esophageal adenocarcinoma (EAC), stratified by [file CAM4-5-2657-s001.docx]

| **Supplementary Table S1. Study sample flow-chart** | | | | | | | | | | | | |
| --- | --- | --- | --- | --- | --- | --- | --- | --- | --- | --- | --- | --- |
|  | **GERD (N)** | | | **BE (N)** | | | **EAC (N)** | | | **Controls (N)** | | |
| **Study (reference number)** | **Eligible** | **Requested** | **Analysed** | **Eligible** | **Requested** | **Analysed** | **Eligible** | **Requested** | **Analysed** | **Eligible** | **Requested** | **Analysed** |
| SRD (12) | 395 | 200 | 200 | 160 | 100 | 100 | - | | | 167 | 100 | 100 |
| FINBAR (13) | 230 | 200 | 186^b^ | 199 | 100 | 100 | 198 | 200 | 58^bc^ | 220 | 100 | 91^a^ |
| SDH (14) | - | | | 329 | 104 | 104 | - | | | 328 | 104 | 104 |
| EIBO (15) | - | | | 244 | 100 | 99 | - | | | 209 | 100 | 100 |
| LAM (16) | - | | | - | | | 60 | 53 | 53 | 448 | 53 | 53 |
| ACS(17) | - | | | - | | | 244 | 226 | 226 | 245 | 242 | 242 |
| SECC (18) | - | | | - | | | 65 | 50 | 47^a^ | 124 | 50 | 46^a^ |
| Total samples analyzed | 386 | | | 403 | | | 384 | | | 736 | | |
| ^a^ Samples missing due to insufficient amount of DNA, ^b^ DNA was purified at different labs with various methods, ^c^35 cases were misclassified Abbreviation: GERD, gastroesophageal reflux; BE, Barrett’s esophagus; EAC, esophageal adenocarcinoma; FINBAR, the Factors Influencing the Barrett’s/Adenocarcinoma Relationship study; EIBO, the Epidemiology and Incidence of Barrett’s Oesophagus study; SECC, the Swedish Esophageal and Cardia Cancer study; ACS, the Australian Cancer study; SDH, the Study of Digestive Health; LAM, the Los Angeles Multi-ethnic Study; SRD, the Study of Reflux Disease | | | | | | | | | | | | |

| **Supplementary Table S2. qPCR reaction ^a^** | | | |
| --- | --- | --- | --- |
| **Reaction** | **Primer** | **Sequence** | **Reaction condition** |
| qPCR | tel1b | CGGTTTGTTTGGGTTTGGGTTTGGGTTTGGGTTTGGGTT | Denaturation: 15min at 95°C  Amplification: 7s at 95°C and 10s at 56°C  N° of cycles: 30 cycles |
|  | tel2b | GGCTTGCCTTACCCTTACCCTTACCCTTACCCTTACCCT |  |
| control-gene | 36B4u | CAGCAAGTGGGAAGGTGTAATCC | Denaturation: 15min at 95°C  Amplification: 7s at 95°C and 20s at 56°C  N° of cycles: 35 cycles |
|  | 36B4d | CCCATTCTATCATCAACGGGTACAA |  |
| ^a^ All PCR reactions were set up with a QIAgility pipetting robot (Qiagen, Hilden, Germany) and performed in 100-well using Rotor Gene Q (Qiagen, Hilden, Germany). Each reaction included 1×Rotor Gene Sybrgreen PCR master mix (Qiagen, Hilden, Germany), 2.5ng of DNA, 500nM primer and 1uM control-gene primer. In addition, 400nM of a passive HEX-labeled oligo was include in all reactions as a passive reference dye. The Rotor Gene software (Qiagen, Hilden, Germany) was used to normalize the Sybrgreen intensity to the HEX passive reference dye. | | | |

| **Supplementary Table S3. Study characteristics of GERD, BE and EAC cases by study** | | | | | | | | | | | | | | | | | | | | |
| --- | --- | --- | --- | --- | --- | --- | --- | --- | --- | --- | --- | --- | --- | --- | --- | --- | --- | --- | --- | --- |
|  | **GERD (w/o BE or EA)** | | | | | |  | **BE** | | | | | | |  | **EAC** | | | | |
| **Study** | **N** | **Sex, male (%)** | **Age, mean (SD)** | **BMI, mean kg/m^2^ (SD)** | **Smoking, ever (%)** | **Telomere length median (IQR)** |  | **N** | **Sex, male (%)** | **Age, mean (SD)** | **BMI, mean kg/m^2^ (SD)** | **Smoking, ever (%)** | **Telomere length median (IQR)** |  | **N** | **Sex, male (%)** | **Age, mean (SD)** | **BMI, mean kg/m^2^ (SD)** | **Smoking, ever (%)** | **Telomere length median (IQR)** |
| SRD (12) ^a^ | 200 | 105 (53) | 50.5 (12.9) | 28.9 (6.0) | 98 (49) | 1.12 (0.94-1.26) |  | 100 | 66 (66) | 55.0 (12.4) | 29.8 (5.5) | 67 (67) | 1.07 (0.89-1.21) |  |  |  |  |  |  |  |
| FINBAR (13) ^b^ | 186 | 156 (84) | 61.7 (11.6) | 29.2 (4.1) | 99 (53) | 0.87 (0.76-1.00) |  | 100 | 82 (82) | 62.0 (11.8) | 28.4 (4.2) | 60 (60) | 0.84 (0.72-0.93) |  | 58 | 48 (83) | 64.6 (11.2) | 28.2 (4.5) | 46 (79) | 0.78 (0.71-0.98) |
| SDH (14) ^c^ |  |  |  |  |  |  |  | 104 | 70 (67) | 61.3 (10.2) | 28.1 (4.7) | 73 (70) | 0.89 (0.81-0.99) |  |  |  |  |  |  |  |
| EIBO (15) ^b^ |  |  |  |  |  |  |  | 99 | 80 (81) | 62.8 (10.8) | 30.2 (5.7) | 71 (72) | 1.00 (0.89-1.15) |  |  |  |  |  |  |  |
| LAM (16) ^d^ |  |  |  |  |  |  |  |  |  |  |  |  |  |  | 53 | 47 (89) | 60.2 (9.1) | 27.6 (4.8) | 41 (77) | 0.71 (0.62-0.84) |
| ACS (17) ^c^ |  |  |  |  |  |  |  |  |  |  |  |  |  |  | 226 | 211 (93) | 63.4 (9.2) | 29.7 (5.2) | 167 (74) | 0.92 (0.82-1.07) |
| SECC (18) ^b^ |  |  |  |  |  |  |  |  |  |  |  |  |  |  | 47 | 43 (91) | 66.2 (8.6) | 27.0 (4.0) | 23 (55) | 0.87 (0.78-0.97) |
| Total | 386 | 261 (68) | 55.9 (13.6) | 29.0 (5.1) | 197 (51) | 0.96 (0.83-1.16) |  | 403 | 298 (74) | 60.3 (11.7) | 29.1 (5.1) | 271 (67) | 0.94 (0.82-1.08) |  | 384 | 349 (91) | 63.5 (9.6) | 28.8 (5.0) | 280 (73) | 0.88 (0.76-1.02) |
| ^a^ 5-Prime (5-Prime, Hilden, Germany), ^b^ Gentra Puregene DNA purification kit (Qiagen, Hilden, Germany), ^c^ Protein Salting Out, ^d^ Qiagen QIAamp DNA Blood kit (Qiagen, Hilden, Germany) Abbreviation: GERD, gastroesophageal reflux; BE, Barrett’s esophagus; EAC, esophageal adenocarcinoma; FINBAR, the Factors Influencing the Barrett’s/Adenocarcinoma Relationship study; EIBO, the Epidemiology and Incidence of Barrett’s Oesophagus study; SECC, the Swedish Esophageal and Cardia Cancer study; ACS, the Australian Cancer study; SDH, the Study of Digestive Health; LAM, the Los Angeles Multi-ethnic Study; SRD, the Study of Reflux Disease; IQR, interquartile range; BMI, body mass index | | | | | | | | | | | | | | | | | | | | |

| **Supplementary Table S4.**  **Leukocyte telomere length and prevalence of GERD, BE and EAC stratified by smoking, obesity and alcohol consumption** | | | | | | | | | | | | | | | | | | | | | | | | |
| --- | --- | --- | --- | --- | --- | --- | --- | --- | --- | --- | --- | --- | --- | --- | --- | --- | --- | --- | --- | --- | --- | --- | --- | --- |
|  |  | **Ever smokers** | | |  | **Non-smokers** | | |  | **BMI <30 kg/m^2^** | | |  | **Obese BMI >30 kg/m^2^** | | |  | **Consumers of alcohol** | | |  | **Non-consumers of alcohol** | | |
|  | **LTL** | **Control** | **Case** | **OR (95%CI)^a^** |  | **Control** | **Case** | **OR (95%CI) ^a^** |  | **Control** | **Case** | **OR (95%CI) ^a^** |  | **Control** | **Case** | **OR (95%CI) ^a^** |  | **Control** | **Case** | **OR (95%CI) ^a^** |  | **Control** | **Case** | **OR (95%CI) ^a^** |
| **GERD vs. population based controls** | 1st (Long) | 40 | 91 | 1 [Reference] |  | 37 | 86 | 1 [Reference] |  | 60 | 120 | 1 [Reference] |  | 16 | 56 | 1 [Reference] |  | 65 | 148 | 1 [Reference] |  | 12 | 29 | 1 [Reference] |
| Studies included; FINBAR, SRD | 2^nd^ | 24 | 57 | 1.07 (0.56-2.03) |  | 25 | 55 | 0.81 (0.42-1.53) |  | 34 | 79 | 1.08 (0.64-1.84) |  | 15 | 33 | 0.40 (0.15-1.07) |  | 40 | 85 | 0.81 (0.49-1.34) |  | 9 | 27 | 1.43 (0.49-4.15) |
|  | 3rd (Short) | 39 | 49 | 0.55 (0.29-1.05) |  | 24 | 48 | 0.56 (0.27-1.18) |  | 48 | 61 | 0.56 (0.31-0.98) |  | 17 | 35 | 0.44 (0.15-1.23) |  | 47 | 65 | 0.48 (0.27-0.85) |  | 18 | 30 | 0.93 (0.34-2.51) |
|  | continuous |  |  | 0.35 (0.11-1.10) |  |  |  | 0.34 (0.08-1.39) |  |  |  | 0.56 (0.21-1.55) |  |  |  | 0.05 (0.01-0.43) |  |  |  | 0.34 (0.12-0.95) |  |  |  | 0.46 (0.07-3.07) |
|  | *P*_trend_ |  |  | 0.07 |  |  |  | 0.13 |  |  |  | 0.27 |  |  |  | 0.01 |  |  |  | 0.04 |  |  |  | 0.42 |

| **BE vs. population based controls** | 1st (Long) | 73 | 99 | 1 [Reference] |  | 69 | 54 | 1 [Reference] |  | 103 | 89 | 1 [Reference] |  | 38 | 64 | 1 [Reference] |  | 115 | 121 | 1 [Reference] |  | 27 | 32 | 1 [Reference] |
| --- | --- | --- | --- | --- | --- | --- | --- | --- | --- | --- | --- | --- | --- | --- | --- | --- | --- | --- | --- | --- | --- | --- | --- | --- |
| Studies included; SDH, EIBO, FINBAR, SRD | 2^nd^ | 66 | 101 | 1.22 (0.77-1.94) |  | 60 | 38 | 0.73 (0.42-1.29) |  | 93 | 81 | 0.93 (0.61-1.43) |  | 33 | 57 | 1.09 (0.59-2.01) |  | 108 | 104 | 0.85 (0.58-1.26) |  | 18 | 35 | 1.66 (0.77-3.61) |
|  | 3rd (Short) | 79 | 71 | 0.73 (0.45-1.17) |  | 46 | 40 | 0.79 (0.43-1.47) |  | 91 | 72 | 0.76 (0.48-1.21) |  | 34 | 37 | 0.68 (0.35-1.31) |  | 97 | 85 | 0.76 (0.50-1.17) |  | 30 | 25 | 0.69 (0.31-1.53) |
|  | continuous |  |  | 0.76 (0.31-1.86) |  |  |  | 0.46 (0.14-1.45) |  |  |  | 0.86 (0.37-1.98) |  |  |  | 0.21 (0.05-0.89) |  |  |  | 0.81 (0.37-1.75) |  |  |  | 0.26 (0.05-1.42) |
|  | *P*_trend_ |  |  | 0.55 |  |  |  | 0.18 |  |  |  | 0.72 |  |  |  | 0.03 |  |  |  | 0.59 |  |  |  | 0.12 |
| **EAC vs. population based controls** | 1st (Long) | 72 | 75 | 1 [Reference] |  | 47 | 31 | 1 [Reference] |  | 101 | 64 | 1 [Reference] |  | 18 | 39 | 1 [Reference] |  | 109 | 99 | 1 [Reference] |  | 10 | 7 | 1 [Reference] |
| Studies included; SDH, EIBO, FINBAR, SRD  Studies included; ACS, LAM, FINBAR, SECC | 2^nd^ | 89 | 92 | 1.05 (0.67-1.64) |  | 54 | 32 | 0.75 (0.45-1.62) |  | 106 | 84 | 1.18 (0.76-1.80) |  | 36 | 38 | 0.45 (0.22-0.93) |  | 131 | 106 | 0.89 (0.61-1.30) |  | 12 | 18 | 2.20 (0.64-7.58) |
|  | 3rd (Short) | 100 | 113 | 1.19 (0.75-1.88) |  | 68 | 39 | 0.72(0.37-1.39) |  | 140 | 104 | 1.04 (0.68-1.61) |  | 26 | 47 | 0.70 (0.32-1.54) |  | 137 | 124 | 1.01 (0.68-1.51) |  | 33 | 28 | 1.32 (0.42-4.14) |
|  | continuous |  |  | 1.98 (0.73-5.37) |  |  |  | 0.78 (0.21-2.99) |  |  |  | 1.66 (0.66-4.18) |  |  |  | 0.57 (0.11-2.80) |  |  |  | 1.45 (0.62-3.37) |  |  |  | 1.34 (0.14-12.8) |
|  | *P*_trend_ |  |  | 0.18 |  |  |  | 0.72 |  |  |  | 0.29 |  |  |  | 0.49 |  |  |  | 0.39 |  |  |  | 0.8 |
| **BE vs. GERD**  Studies included; FINBAR, SRD | 1st (Long) | 91 | 45 | 1 [Reference] |  | 86 | 30 | 1 [Reference] |  | 120 | 44 | 1 [Reference] |  | 56 | 31 | 1 [Reference] |  | 148 | 60 | 1 [Reference] |  | 29 | 15 | 1 [Reference] |
|  | 2^nd^ | 57 | 41 | 1.55 (0.87-2.76) |  | 55 | 20 | 0.88 (0.43-1.79) |  | 79 | 36 | 1.21 (0.70-2.10) |  | 33 | 24 | 1.40 (0.65-2.98) |  | 85 | 43 | 1.33 (0.80-2.22) |  | 27 | 18 | 1.09 (0.45-2.69) |
|  | 3rd (Short) | 49 | 41 | 1.86 (0.98-3.52) |  | 48 | 23 | 0.98 (0.45-2.13) |  | 61 | 44 | 1.82 (1.00-3.29) |  | 35 | 20 | 0.90 (0.38-2.15) |  | 65 | 46 | 1.92 (1.08-3.40) |  | 30 | 17 | 0.79 (0.31-2.02) |
|  | continuous |  |  | 6.02 (1.79-20.2) |  |  |  | 0.34 (0.09-1.35) |  |  |  | 2.11 (0.66-6.78) |  |  |  | 1.41 (0.35-5.62) |  |  |  | 3.46 (1.18-10.1) |  |  |  | 0.46 (0.09-2.32) |
|  | *P*_trend_ |  |  | 0.01 |  |  |  | 0.13 |  |  |  | 0.21 |  |  |  | 0.63 |  |  |  | 0.02 |  |  |  | 0.35 |
| **^a^** Minimally adjusted: adjusted for age, sex and DNA extraction method. Abbreviation: LTL, leukocyte telomere length ;OR, Odds Ratio; CI, Confidence Interval; BMI, body mass index; GERD, gastroesophageal reflux; BE, Barrett’s esophagus; EAC, esophageal adenocarcinoma; FINBAR, the Factors Influencing the Barrett’s/Adenocarcinoma Relationship study; EIBO, the Epidemiology and Incidence of Barrett’s Oesophagus study; SECC, the Swedish Esophageal and Cardia Cancer study; ACS, the Australian Cancer study; SDH, the Study of Digestive Health; LAM, the Los Angeles Multi-ethnic Study; SRD, the Study of Reflux Disease | | | | | | | | | | | | | | | | | | | | | | | | |
